# Therapeutic potential and action mechanisms of licochalcone B: a mini review

**DOI:** 10.3389/fmolb.2024.1440132

**Published:** 2024-07-03

**Authors:** Sibhghatulla Shaikh, Eun Ju Lee, Khurshid Ahmad, Inho Choi

**Affiliations:** ^1^ Department of Medical Biotechnology, Yeungnam University, Gyeongsan, Republic of Korea; ^2^ Research Institute of Cell Culture, Yeungnam University, Gyeongsan, Republic of Korea

**Keywords:** flavonoid, licochalcone B, anti-cancer, anti-inflammatory, hepatoprotective

## Abstract

Licochalcone B (LicB), a chalcone derived from *Glycyrrhiza uralensis* and *Glycyrrhiza* glabra, has received considerable attention due to its diverse pharmacological properties. Accumulated data indicates that LicB has pharmacological effects that include anti-cancer, hepatoprotective, anti-inflammatory, and neuroprotective properties. The action mechanism of LicB has been linked to several molecular targets, such as phosphoinositide 3-kinase/Akt/mammalian target of rapamycin, p53, nuclear factor-κB, and p38, and the involvements of caspases, apoptosis, mitogen-activated protein kinase-associated inflammatory pathways, and anti-inflammatory nuclear factor erythroid 2–related factor 2 signaling pathways highlight the multifaceted therapeutic potential of LicB. This review systematically updates recent findings regarding the pharmacological effects of LicB, and the mechanistic pathways involved, and highlights the potential use of LicB as a promising lead compound for drug discovery.

## 1 Introduction

The pharmacological properties of various herbal preparations and natural compounds have been the subjects of increasing research in recent years (Atanasov et al., 2021; Shaikh et al., 2021; Ahmad et al., 2023). *Glycyrrhiza* spp. (belonging to Fabaceae family), also called licorice, sweet wood, or mulaithi, is a perennial herb found globally. The name combines the Greek words “glykys” (sweet) and “rhiza” (root) (El-Saber Batiha et al., 2020). *Glycyrrhiza* spp. contains a variety of bioactive compounds including chalcones with various biological properties that include anti-inflammatory, antiviral, antimicrobial, antioxidant, anticancer, immunomodulatory, hepatoprotective, and cardioprotective effects (Asl and Hosseinzadeh, 2008; Yang et al., 2015). Chalcones and their derivatives have attracted considerable interest in the medical field due to their wide-ranging pharmacological activities and clinical potential. At the molecular level, chalcones contain two aromatic rings linked by an α, β-unsaturated α-carbon ketone moiety, which forms the core structure (Zhuang et al., 2017). Pharmacologically, licochalcone A and licochalcone B (LicB), which are components of the roots of *Glycyrrhiza* spp., have emerged as noteworthy bioactive compounds. The pharmacological effects of licochalcone A have recently been reviewed extensively elsewhere (Li M. T. et al., 2022), and thus, here we focus on the pharmacology of LicB.

LicB has a variety of pharmacological effects; for example, it is cardioprotective (Han et al., 2014), has anti-Alzheimer’s disease effects (Cao et al., 2020), possesses anti-oxidant and radical scavenging properties (Fu et al., 2013), and the ability to induce cancer cell apoptosis (Yuan et al., 2014; Yu et al., 2016). Our research group continues to search for naturally occurring compounds that target proteins involved in skeletal muscle (SM) and associated disorders (Baig et al., 2017; Ahmad et al., 2021; Lee et al., 2021; Ali et al., 2022; Ali et al., 2023a; Ali et al., 2023b). Recently, we demonstrated that LicB binds strongly to the catalytic site of DPP4 and concentration-dependently inhibits its activity, which suggests LicB has potential use as an antidiabetic agent (Shaikh et al., 2022). The promising therapeutic potential of LicB for the management and prevention of clinical diseases emphasizes the need for an updated review of its pharmacology. Thus, this review was undertaken to summarize recent pharmacologic advances, investigate the potential applications of LicB for the management of various diseases, and provide critical insight into the clinical use of LicB. In addition, we discuss the toxicity profile of LicB.

## 2 Pharmacological effects

### 2.1 Effects on skeletal muscle/myogenesis

SM is the most abundant body tissue, and in addition to body movements and posture, it regulates temperature and physically protects internal organs and soft tissues. In addition, SM can regenerate in response to injury or disease and is an important marker of diabetes, obesity, and age-related diseases. Recently, we found that *Glycyrrhiza uralensis* inhibits MSTN, a key (negative) regulator of muscle growth, and promotes myogenesis and that LicB is a primary promoter of myoblast differentiation and proliferation (Lee et al., 2021). This study provided a basis for a more comprehensive investigation of the LicB’s biologic mechanisms of LicB, especially on its ability to regulate MSTN and atrogin1 and MuRF1 (markers of muscle atrophy) employing *in silico*, *in vitro*, and *in vivo* approaches. A subsequent study revealed how LicB inhibits MSTN and promotes muscle proliferation and differentiation, which are both required for muscle regeneration. These studies improved our understanding of the therapeutic mechanisms of LicB, its anti-aging properties, and its ability to ameliorate muscle-wasting conditions. These findings enhanced the therapeutic narrative and established LicB as a clinical candidate (Ahmad et al., 2024).

### 2.2 Anti-inflammatory effects

The abnormal activation of NLRP3 (NLR family pyrin domain containing 3) inflammasome has been linked to a variety of inflammatory pathologies, and LicB inhibits NLRP3 inflammasome activation in macrophages. The mechanism involves LicB binding directly to NEK7, thus disrupting the interaction between NLRP3 and NEK7 and effectively suppressing the activation of NLRP3 inflammasome. Additionally, in murine models, LicB protected against NLRP3-mediated diseases such as non-alcoholic steatohepatitis, lipopolysaccharide (LPS) and monosodium urate stimulated septic shock, and peritonitis (Li Q. et al., 2022). Collectively, LicB appears to be a selective NLRP3 inhibitor with therapeutic potential for the treatment of NLRP3 inflammasome-related disorders.

LicB has been demonstrated to suppress PKA activation effectively, and thus, to inhibit LPS-stimulated NF-κB phosphorylation at serine 276 and activation in RAW264.7 cells. Furthermore, LicB significantly reduced LPS-stimulated nitric oxide (NO), TNF-α, and MCP-1 generation in RAW264.7 cells (Furusawa et al., 2009). Similarly, in another study, LicB exhibited potent inhibitory activity against LPS-induced NO production in RAW 264.7 cells with an IC_50_ of 8.78 μM (Lin et al., 2017b). In addition, LicB attenuated BDE-47-stimulated oxidative damage in RAW264.7 cells by activating the Nrf2 pathway and inhibiting the NF-κB pathway, thereby preventing immunological dysfunction and apoptosis (Dong M. et al., 2024).

LicB also potently scavenged ABTS(+) radicals and inhibited lipid peroxidation in rat liver microsomes, dose-dependently suppressed ROS generation in RAW264.7 cells, and effectively inhibited NO, IL-6, and prostaglandin E2 generation in LPS-stimulated macrophages (Fu et al., 2013).

The enzyme 15-lipoxygenase (15-LOX) is a critical target for treating various inflammatory disorders, and LicB inhibits 15-LOX with an IC_50_ of 9.67 μM (Li et al., 2021). Furthermore, LicB inhibits TNF-α release by LPS-induced RAW264.7 cells. Computational analysis demonstrated that the targeting of Thr412, Arg415, Val420, Thr429, Ile602, and Trp606 of 15-LOX by LicB was largely responsible for 15-LOX inhibition (Li et al., 2021). This study identified LicB as a 15-LOX inhibitor and laid the groundwork for future 15-LOX inhibitor research.

LicB reduced inflammation and oxidative stress in LPS-stimulated periodontal ligament cells. LicB inhibited the NF-κB/NLRP3 signaling pathway and reversed LPS-induced inhibition of osteogenic differentiation. These findings suggest that LicB has the potential to be a periodontal therapeutic agent (Du et al., 2024).

### 2.3 Neuroprotective effects

Interestingly, LicB was also found to ameliorate memory impairments and neurological deficits in a mouse Middle Cerebral Artery Occlusion (MCAO) model of stroke. Histological examinations revealed that LicB-mediated improvements in neuronal injury and apoptosis were accompanied by the downregulation of apoptosis-related proteins. Furthermore, LicB attenuated post-MCAO reductions in brain-derived neurotrophic and nerve growth factor levels. Furthermore, LicB suppressed oxidative stress and inflammation, as evidenced by increased SOD and GSH levels, elevated IL-4 levels, and reduced malondialdehyde (MDA), iNOS (inducible nitric oxide synthase), and TNF-α levels (Zhou et al., 2021).

Qu et al. (2022) investigated the neuroprotective abilities of LicB on oxidative stress-stimulated damage in neural cells. H_2_O_2_ was used to simulate ROS-induced cell apoptosis in PC-12 cells, and in these stimulated cells, LicB reduced apoptosis and cell cytotoxic and enhanced the levels of apoptosis-related proteins such as caspase-3 and cleaved caspase-3. Notably, LicB effectively reduced MDA, SOD, and ROS in H_2_O_2_-induced cells. In the same study, LicB inhibited apoptosis by activating ATG7-dependent autophagy and the SIRT1/AMPK signaling pathway, as demonstrated by an increase in LC3-II levels and a reduction in p62 levels in neural cells and *Caenorhabditis elegans* models. Altogether, LicB has exhibited promising neuroprotective effects in models of oxidative damage and has been found to employ a variety of pharmacological mechanisms.

LicB has also been shown to inhibit the self-aggregation of Aβ_42_ and to disassemble pre-formed Aβ_42_ fibrils. Furthermore, LicB reduces chelating metal ion-induced Aβ_42_ aggregation. In silico analysis showed that LicB inhibits Aβ_42_ self-aggregation by forming two H-bonds with Lys28 and obstructing the salt bridge interaction at the C-terminus of Aβ_42_. Furthermore, LicB exhibits neuroprotective properties in SH-SY5Y cells against H_2_O_2_-induced cell death (Cao et al., 2020).

### 2.4 Anti-cancer effects

LicB had a dose-dependent inhibitory effect on the growth of osteosarcoma (OS) cells, specifically MG-63 and U2OS cells. In these cells, LicB induced autophagy and apoptosis and, at the molecular level, reduced Bcl-2, p62, caspase-3, and Ki67 protein levels but increased cleaved caspase-3, Beclin1, Bax, Atg7, and LC3B levels, and thus, promoted apoptosis. Furthermore, LicB inhibited the PI3K/AKT/mTOR pathway and induced autophagy in MG-63 and U2OS cells (Huang and Jin, 2022). These findings shed light on the intricate molecular mechanisms underlying the anti-cancer effects of LicB in osteosarcoma.

Wang et al. reported LicB inhibited HepG2 cell growth (IC_50_ = 110.15 μM), causing morphological distortions, cell cycle arrest at the G2/M phase, apoptosis, and accompanying intracellular ROS generation. LicB also significantly altered mRNA and protein expressions of the cell cycle-related genes. Notably, the inhibitions of caspase 8 and 9 reduced LicB-stimulated apoptosis (Wang et al., 2019).

In addition, the transcriptome and small RNA levels of LicB-responsive mRNAs and miRNAs in HepG2 cells have been investigated. A thorough examination revealed 85 differentially expressed miRNAs (DEMs) and 763 differentially expressed genes (DEGs) and that DEGs in LicB-treated cells were significantly enriched in components of the MAPK signaling pathway, a key regulator of hepatocellular carcinoma. Two key DEMs, namely, hsa-miR-29b-3p and has-miR-96-5p, were identified. These findings emphasized the importance of understanding the regulatory mechanisms of LicB in HepG2 cells and confirmed its potential as a functional dietary ingredient in liver cancer therapy. Understanding of the potential anti-tumor effects of LicB was also improved by omics data suggesting that miRNA and mRNA expression patterns might serve as promising biomarkers for identifying novel therapeutic targets in liver cancer (Wang and Wang, 2021).

LicB significantly inhibited oral squamous cell carcinoma (SCC) cell proliferation, particularly HN22 and HSC4 cells. LicB arrested the cell cycle at G1, downregulating cyclin D1 while upregulating the expression of p21 and p27 proteins. Additionally, LicB translocated phospholipid phosphatidylserine from the inner to outer leaflets of the plasma membrane, causing chromatin condensation, DNA fragmentation, and sub-G1 cell accumulation. LicB also increased ROS production, which induced CHOP, death receptor (DR) 4, and DR5. Furthermore, anti-apoptotic proteins (Bid, Bcl-xl, and Mcl-1) were downregulated, while the pro-apoptotic protein (Bax) was upregulated by LicB, and LicB-induced MMP loss resulted in the release of cytochrome c. Apoptotic protease activating factor-1 and survivin expression were also altered to promote apoptosis (Oh et al., 2016). Altogether, LicB shows significant promise as a therapeutic agent for managing human oral cancer by inducing apoptotic cell death.

In hepatocellular carcinoma cells, LicB inhibited proliferation and induced DNA damage, cell cycle arrest, and apoptosis. LicB inhibited the AKT/mTOR pathway and simultaneously activated ER stress and the MAPK signaling pathway. Furthermore, in these cells, co-treatment with LicB and TRAIL (TNF-related apoptosis-inducing ligand) significantly enhanced LicB-induced viability reduction and apoptosis. LicB also increased the protein expressions of DR4 and DR5. On the other hand, treatment with PD98059 or SP600125 (ERK inhibitor and JNK inhibitors, respectively) significantly decreased the LicB-stimulated upregulation of DR5 expression and attenuated LicB-mediated TRAIL sensitization (Zhang et al., 2022).

In a recent study, Kwak et al. (2023) investigated the anti-tumor efficacy of LicB against human colorectal cancer (CRC) and the underlying molecular mechanisms involved. LicB treatment significantly decreased cell viability, increased ROS generation, mitochondrial membrane potential (MMP) depolarization, caspase activation, and JNK/p38 MAPK signaling pathway activations, and induced apoptosis and G2/M cell cycle arrest. However, pretreatment with SB203580 or SP600125 (p38 and JNK inhibitors, respectively) suppressed LicB-stimulated reductions in cell viability. In addition, the ROS scavenger N-acetylcysteine effectively prevented the effects of LicB on tumor cell viability, apoptosis, ROS production, cell cycle arrest, MMP depolarization, and upregulations of the activities of caspases and JNK/p38 MAPK. This study demonstrated LicB has therapeutic potential in CRC.

Sadek et al. (2020) investigated the combined effects of LicB and fullerene C_60_ (FnC_60_) nanoparticles on diethylnitrosamine-induced hepatocarcinoma *in vivo*. Treatment with LicB alone or in combination with FnC_60_ reduced diethylnitrosamine-induced DNA fragmentation and oxidative DNA damage and downregulated the mRNA expressions of APE1/Ref-1, CDK-4, retinoblastoma, Bcl-2, B-cell lymphoma-xL, and β-arrestin-2 and the protein expressions of APE1/Ref-1 and CDK-4. Furthermore, these LicB-induced changes increased the protein expressions of p53 protein, Bcl-2-associated X, and caspase-3.

In addition, LicB inhibited esophageal SCC growth by directly inhibiting the activity of JAK2 and its subsequent signaling pathway. LicB also inhibited the growth of KYSE450 and KYSE510 esophageal SCC cells by arresting the cell cycle at the G2/M phase and increasing apoptosis. Notably, the effect of AZD1480 (a JAK2 inhibitor) on esophageal SCC cells was comparable to that of LicB. Furthermore, LicB reduced the phosphorylation of STAT3 at Y705 and S727 and the expression of Mcl-1 (a target of STAT3) (Song et al., 2020). These findings point to the potential therapeutic role of LicB for managing esophageal SCC based on targeting the JAK2 signaling pathway.

LicB was also found to inhibit the proliferation of SCC and human melanoma cells by causing apoptotic cell death through specificity protein 1 (transcription factor) and other apoptosis-related proteins, including CCAAT/enhancer-binding protein homologous protein, DRs, and poly (ADP-ribose) polymerase (PARP) (Kang et al., 2017).

In human non-small cell lung cancer (NSCLC) cells, Oh et al. (2019) found that LicB inhibited the activities of EGFR and MET kinase and cell viability in HCC827 and HCC827GR cells. In addition, LicB stimulated G2/M cell-cycle arrest and apoptosis, caused ER stress, ROS production, and MMP loss, and activated caspases. Also, the inhibitions of EGFR and MET reduced activation of the ERBB3/AKT axis. These observations indicate LicB is a promising, novel therapeutic candidate for the treatment of NSCLC.

LicB inhibited proliferation, caused S phase arrest, reduced cyclin A, CDK1, and CDK2 levels and the expression of cell division cycle 25 (Cdc25A and Cdc25B) in T24 EJ human bladder cancer cells, upregulated the expressions of Bcl-2, survivin, and Bax expression, and caused PARP cleavage. LicB also markedly reduced the tumorigenicity of LicB-treated MB49 cells *in vitro*, and this finding was supported by *in vivo* studies on an MB49 mouse model of bladder cancer (Yuan et al., 2014). Similarly, in another study, LicB inhibited the cell migration, adhesion, and invasion of T24 cells, inhibited the expressions of MMP-9 mRNA and protein, reduced NF-кB p65 levels, and impeded the nuclear translocation of NF-кB (Zhao et al., 2014).

**FIGURE 1:**
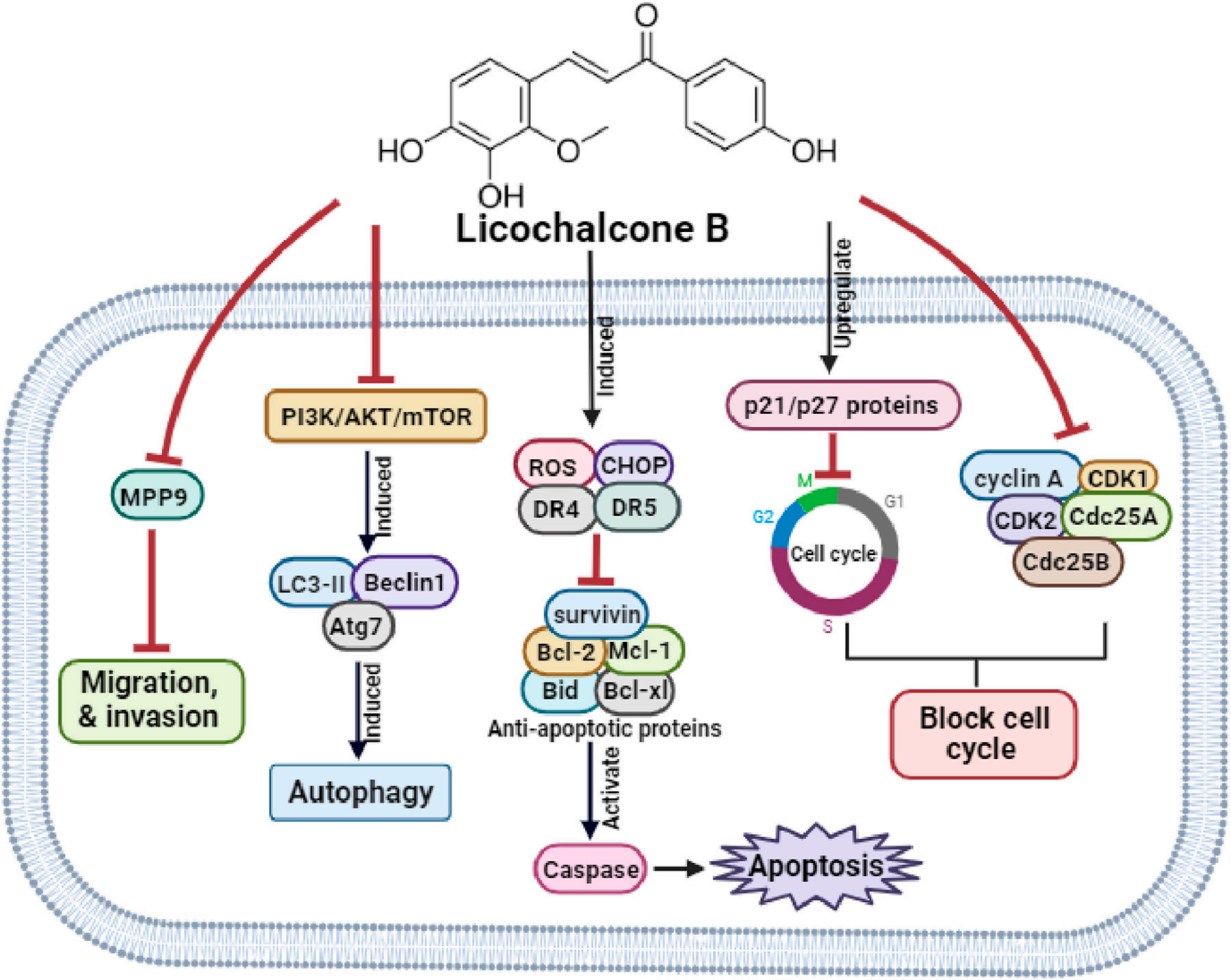
The molecular mechanisms underlying LicB’s anti-cancer effects.

Yu et al. (2016) reported that LicB inhibited the proliferation of MCF-7 breast cancer cells using a mechanism involving S phase arrest attributed to decreases in Cyclin A, Cdk2, and Cdc25 A levels and an increase in p21 protein levels and that it induced apoptosis, as evidenced by morphological changes including phosphatidylserine externalization and DNA fragmentation. LicB also disrupted MMP causing cytochrome C release, and molecular analysis revealed that LicB increased the expressions of Caspase-3, Caspase-9, and Bax expression but reduced Bcl-2 expression. Furthermore, these effects were attributed to an increased p53 protein level in LicB-treated cells. Collectively, LicB has potential use as a therapeutic agent for the management of breast cancer. The anti-cancer molecular mechanisms of LicB are depicted in Figure 1.

### 2.5 Cardioprotective effects

ROS production is a major contributor to ischemia-reperfusion-induced cardiac injury. In a study by Han et al. (2014), pretreatment with LicB improved heart rate, left ventricular developed pressure, and reduced creatine kinase and LDH levels in coronary flow in Sprague-Dawley rat model. LicB treatment also increased SOD levels and GSH/GSSG ratios and reduced IL-8 and IL-6 activities and MDA, TNF-α, and C-reactive protein (CRP) levels. Furthermore, LicB-treated group had smaller infarct size and exhibited less apoptosis than ischemia/reperfusion control group. The authors suggested that its cardioprotective effects were probably due to its antioxidative, anti-apoptotic, and anti-inflammatory properties. In another study, LicB potently inhibited angiotensin-converting enzyme (a critical target in cardiovascular disease) with an IC_50_ value of 0.24 μM (Li et al., 2023).

### 2.6 Hepatoprotective effects

LicB also exhibits hepatoprotective effects against carbon tetrachloride (CCl_4_)-induced injury, which was characterized by elevated ALT, AST, MDA, IL-6, CRP, and TNF-α levels and a reduction in serum SOD and GSH/GSSG ratio. A histopathologic examination of CCl_4_-treated animal liver sections revealed inflammation and necrosis. However, LicB pretreatment prevented these CCl_4_-induced effects, reduced ALT, AST, MDA, GSSG, IL-6, CRP, and TNF-α levels and the protein expressions of p38 and NF-κB but enhanced SOD and GSH levels (Teng et al., 2016). Similarly, in another study, LicB protected against CCl_4_ and acetaminophen-induced hepatocellular injury in HepG2 cells and increased cell viability by more than 80% (Kuang et al., 2017).

Nrf2 protein is a basic-region leucine zipper transcription factor that protects against endogenous and exogenous stressors. Nrf2 increases cellular sensitivity to oxidants and electrophiles by inducing the expressions of various cytoprotective and detoxifying genes. Interestingly, transient Nrf2 activation, facilitated by specific activators, protects against cancer development (Pouremamali et al., 2022), and notably, LicB activation of Nrf2 in HepG2 cells significantly reduced CCl_4_-stimulated acute liver injury in animal model (Lin et al., 2017a).

Oxidative stress is a major contributor to alcohol-induced hepatotoxicity, and LicB reduced ethanol-induced cellular damage and inhibited apoptosis in BRL cells, suggesting a hepatoprotective effect on alcohol-induced cell injury. In addition, LicB markedly reduced ethanol-induced intracellular ROS levels, increased p-Erk expression, and induced the nuclear translocation of Nrf2 (Gao et al., 2017). These results indicate LicB has the potential to treat liver disorders caused by alcohol consumption.

### 2.7 Protective effects on lung injury

**FIGURE 2:**
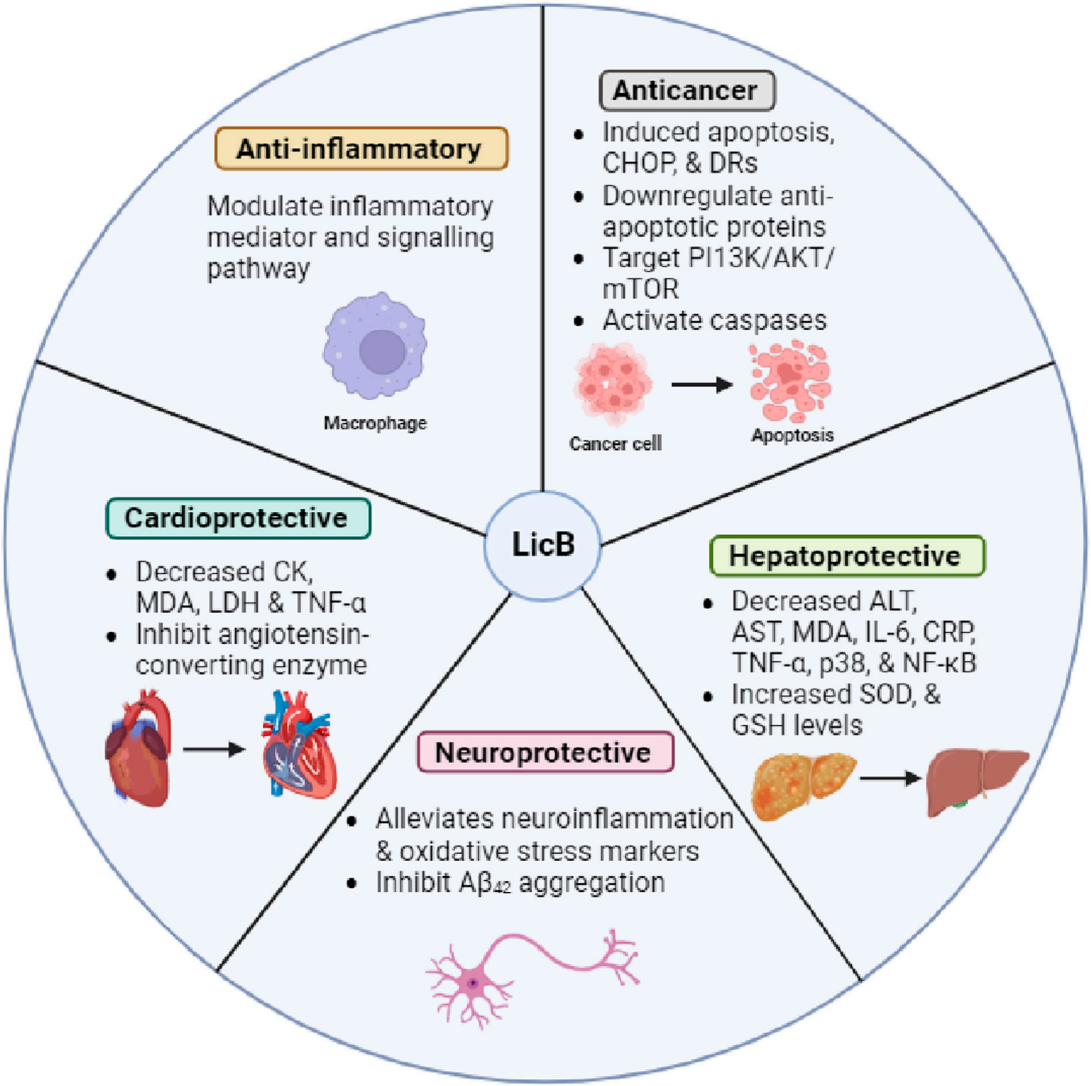
Pharmacological properties of LicB and its underlying mechanisms.

Acute lung injury (ALI) is a severe and often fatal pulmonary disorder. Huang et al. recently explored the effects of LicB on human pulmonary microvascular endothelial cells and LPS-challenged mice. LicB promoted cell viability, had no cytotoxic effect, suppressed apoptosis, and reduced ROS levels. In addition, LicB reduced lung tissue weights, oxidative stress, and inflammatory markers in the ALI murine model. LicB also increased the expressions of Nrf2, HO-1, and NQO1 but decreased the expression of Keap1 (Huang et al., 2023). Collectively, LicB has potential as a therapeutic strategy for alleviating LPS-induced ALI. A summary of the pharmacological properties of LicB is provided in Figure 2.

The optimal combination of LicB and liquiritin showed increased anti-inflammatory, antioxidant, and anti-fibrotic activity. The hemopoietic cell kinase inhibitor (iHCK-37) confirmed that LicB and liquiritin prevent chronic obstructive pulmonary disease (COPD) by inhibiting HCK, resulting in antioxidative, anti-inflammatory, and anti-fibrotic effects. A 1:1 ratio of LicB and liquiritin demonstrated synergistic efficacy in treating COPD by inhibiting HCK activity (Dong S. et al., 2024).

### 2.8 Antiviral effects

LicB has been shown to deactivate herpes simplex virus two particles and prevent viral multiplication via inhibiting Akt phosphorylation and downstream targets. *In vivo* investigations revealed that LicB dramatically lowered viral titers, delayed weight loss, and reduced pathological alterations in vaginal tissues in mouse models of vaginal infection (Li et al., 2024).

### 2.9 Effect in autoimmune diseases

Abnormal activation of the cGAS-stimulator of interferon genes (STING) pathway by abnormal DNA has been linked to autoimmune diseases (Liu and Pu, 2023). LicB inhibited the STING signaling cascade in macrophages by disrupting the STING-TBK1-IRF3 axis and preventing downstream activation. In addition, LicB lowered type I interferon levels in mice treated with the STING agonist CMA and dramatically reduced systemic inflammation in Trex1^−/−^ animals (Luo et al., 2024).

## 3 Toxicity and safety

The toxicological properties of LicB are poorly understood. The enzyme 11β-HSD2 regulates cortisol levels by converting cortisol, an active glucocorticoid hormone, to cortisone, its inactive form (Chapman et al., 2013; Adamidis et al., 2019), and a recent study demonstrated that LicB inhibits human and rat 11β-HSD2 with IC_50_ values of 31.85 μM and 56.56 μM, respectively (Lin et al., 2024).

## 4 Conclusion and future prospective

As described above, LicB has wide-ranging pharmacological activities, which include anti-inflammatory, hepatoprotective, anti-cancer, and neuroprotective effects. These activities are closely related to cellular responses involving inflammation, autophagy, apoptosis, and oxidative stress, which are modulated by signaling pathways such as NF-κβ, SIRT1/AMPK, Nrf2, PI3K/Akt/mTOR, p53, CHOP, DRs, p38, and iNOS, and involve multiple targets such as TNF-α, PI3K, AKT, Bid, Bcl-xl, Mcl-1, and caspases. Hence, we believe that LicB has great promise as a lead compound for drug discovery. Nonetheless, it is essential to remember that most reported LicB findings are based on cellular experiments. Therefore, more clinical research is required to confirm its pharmacological efficacy. Furthermore, its toxicological profile is less well characterized and requires further investigation.

## Abbreviations

LicB, Licochalcone B; SM, Skeletal muscle; NLRP3, NLR family pyrin domain containing 3; LPS, Lipopolysaccharide; NO, Nitric oxide; 15-LOX, 15-lipoxygenase; MDA, Malondialdehyde; OS, Osteosarcoma; DR, Death receptor; FnC60, Fullerene C60; LDH, Lactate dehydrogenase; CCl4, Carbon tetrachloride; ALT, Alanine aminotransferase; AST, Aspartate aminotransferase; Nrf2, Nuclear factor erythroid 2–related factor 2; HCK, Hemopoietic cell kinase; STING, stimulator of interferon genes.
